# Near-Complete Genome Sequence of a Human Norovirus GII.1[Pg] Strain Associated with Acute Gastroenteritis, Determined Using Long-Read Sequencing

**DOI:** 10.1128/MRA.00401-21

**Published:** 2021-07-15

**Authors:** Zhihui Yang, Mark Mammel, Samantha Q. Wales

**Affiliations:** a Division of Molecular Biology, Office of Applied Research and Safety Assessment, Center for Food Safety and Applied Nutrition, U.S. Food and Drug Administration, Laurel, Maryland, USA; KU Leuven

## Abstract

High-throughput sequencing is one of the approaches used for the detection of foodborne pathogens such as noroviruses. Long-read sequencing has advantages over short-read sequencing in speed, read length, and lower fragmentation bias, which makes it a potential powerful tool for the fast detection and identification of viruses. Using Nanopore sequencing technology, we were able to successfully recover a nearly complete genome sequence of a human norovirus GII.1[Pg] strain in a single long read from a sample from a patient with norovirus gastroenteritis.

## ANNOUNCEMENT

Noroviruses are not only the leading cause of foodborne illnesses in the United States but also a primary cause of acute viral gastroenteritis worldwide (1). Noroviruses (genus *Norovirus*, family *Caliciviridae*) can be classified into 10 genogroups (GI to GX) and are further subdivided into at least 48 genotypes. Human noroviruses cluster in genogroups I, II, and IV (2, 3). Due to the high genetic diversity of noroviruses, in addition to PCR-based methods and partial sequencing approaches, high-throughput whole-genome sequencing, which provides nucleotide information on the complete genome, is needed for accurate characterization of these viruses (4, 5). In comparison with short-read sequencing, Nanopore long-read sequencing technologies make it possible to detect and identify viruses faster as well as accurately characterize the genomic complexity with complete coverage of the entire genome by a single read (6). In addition, as a fundamental step during library preparation in short-read sequencing, DNA fragmentation could introduce sequencing bias due to the randomness, incorporation errors, or artifactual indels caused by enzymatic effects (7), and these fragmentation biases can be lowered using Nanopore sequencing technology. Here, we report that using Nanopore sequencing technology and long reads, a nearly complete genome sequence was successfully obtained from a human norovirus GII.1[Pg] strain associated with a case of acute gastroenteritis in the United States in 2016.

A 10% (wt/vol) real-time reverse transcription-PCR (RT-PCR) norovirus-positive stool sample was suspended in phosphate-buffered saline. The supernatant was filtered (via a 4-μm filter), and viral RNA was extracted using a QIAamp viral RNA minikit (Qiagen) (8). Following RNA concentration (Zymo RNA concentration kit), a library was generated using a cDNA-PCR sequencing kit (Nanopore) and sequenced on the GridION platform (Nanopore). Data analysis was performed with the raw sequencing data using our in-house k-mer tool (9) (github.com/mmammel8/kmer_id) and Long Read Support (beta) module within the CLC Genomics Workbench (CLC GWB version 21). All tools were run with default parameters unless otherwise specified. Out of 117,363 total reads, 105,877 were obtained from norovirus, and the average read length was 1,154 bp (ranging from 151 to 7,572 bp). The longest single read (7,572 bp) was used as a BLAST query against the NCBI nucleotide collection database, and this single read covers 98% of the best reference sequence. With *de novo* assembly using the Long Read Support module, one contig covering the norovirus genome sequence was assembled from the 105,877 reads; the average coverage was 16,242×. The genome sequence of Hu GII.1[Pg]/Pennsylvania/2016/USA has a length of 7,521 nucleotides (nt) and a GC content of 47.9% and was annotated via the GenBank norovirus submission tool in the NCBI submission portal. It contains (i) three open reading frames (ORFs), ORF1 (5,099 nt), ORF2 (1,608 nt), and ORF3 (774 nt), and (ii) a 3′ untranslated region (UTR) 61 nt long. The sequence was genotyped as a norovirus GII.1[Pg] strain using both the Norovirus Genotyping Tool version 2.0 (10) and the CaliciNet lab-based Human Calicivirus Typing Tool (https://norovirus.ng.philab.cdc.gov). A full-length genomic sequence BLAST search revealed 96.4% identity with a GII.1[Pg] variant from Indonesia recovered in 2015 (GenBank accession no. MF668937).

### Data availability.

The genome sequence of this GII.1[Pg] strain has been established in GenBank under the accession number MW854326. The raw sequencing data have been deposited in the SRA under the ViroTrakr project (BioProject accession number PRJNA396739 and SRA accession number SRR14119290).
